# Preconception and prenatal vitamin D associations with positive behavioral health in Black children

**DOI:** 10.1017/S0033291724002472

**Published:** 2024-10

**Authors:** Alison E. Hipwell, Kate Keenan, Irene Tung, Allysa Quick, Hyagriv Simhan, Lisa Bodnar, Nia Buckner

**Affiliations:** 1 Department of Psychiatry, University of Pittsburgh, Pittsburgh, PA, USA; 2 Department of Psychology, University of Pittsburgh, Pittsburgh, PA, USA; 3 Department of Psychiatry and Behavioral Neuroscience, University of Chicago, Chicago, IL, USA; 4 Department of Psychology, California State University Dominguez Hills, Carson, CA, USA; 5 Magee-Womens Research Institute and Foundation, Pittsburgh, PA, USA; 6 Department of Epidemiology, School of Public Health, University of Pittsburgh, Pittsburgh, PA, USA; 7 School of Medicine, University of Pittsburgh, PA, USA

**Keywords:** child behavior, positive health, preconception, prenatal, vitamin D

## Abstract

**Background:**

Low levels of vitamin D during pregnancy are associated with offspring behavioral problems but little is known about pre-pregnancy influences. Additionally, Black American individuals are underrepresented in studies, limiting translational impact. We tested independent and interactive effects of preconception and prenatal vitamin D in Black women in relation to positive behavioral and emotional outcomes in early childhood.

**Methods:**

Black-identifying participants (*N* = 156) enrolled in the longitudinal Pittsburgh Girls Study (PGS) provided venous blood samples before and during pregnancy to measure 25-hydroxyvitamin D (25[OH]D) levels. Participants completed questionnaires assessing sociodemographic factors, depression severity and life stress, and later reported on child behavioral and emotional problems and prosocial behavior between 2 and 4 years.

**Results:**

Mean serum 25(OH)D concentrations were 15.5 ng/ml (s.d. = 7.7) before pregnancy and 18.0 ng/ml (s.d. = 9.2) during pregnancy; below the sufficiency threshold according to commonly used dietary guidelines. After adjusting for covariates, prenatal 25(OH)D was negatively related to behavior problems and positively related to prosocial behavior in children, although the association attenuated for behavior problems after accounting for preconception 25(OH)D, which may reflect patterns of stability. Maternal 25(OH)D was unrelated to child emotional problems, and no synergistic effects of 25(OH)D timing were observed for any child outcome.

**Conclusions:**

Findings have relevance for Black women living in the northeast U.S. Results suggest specific associations between maternal vitamin D and positive behaviors in early childhood, regardless of sufficiency levels and suggest potential opportunities for early interventions to support healthy child development.

Vitamin D is an essential micronutrient derived from sunlight, dietary intake and supplements that supports multiple cellular and metabolic processes promoting health throughout the lifespan. The 2020–2025 U.S. Dietary Guidelines (U.S. Department of Agriculture and U.S. Department of Health and Human Services, 2020) define vitamin D levels sufficient for bone health as blood 25-hydroxyvitamin D [25(OH)D] concentrations of 20 to < 50 ng/ml. Although pregnant women are generally considered to be at high deficiency risk (Bodnar et al., 2007; Lee et al., 2007), optimal 25(OH)D concentrations for prenatal health remain uncertain (El-Hajj Fuleihan et al., 2015; Marshall, Mehta, & Petrova, 2013; Maxmen, 2011), partly because those with low levels are often underrepresented in clinical research (Kiely, Wagner, & Roth, 2020). This knowledge gap disproportionately affects Black pregnant women since darker skin pigmentation reduces cutaneous vitamin D synthesis (Ames, Grant, & Willett, 2021; Institute of Medicine, 2011; Webb et al., 2018). Furthermore, some evidence suggests that a lack of prenatal multivitamin use is unrelated to vitamin D insufficiency among Black women (Burris, Thomas, Zera, & McElrath, 2015), raising questions about the utility of commonly used thresholds across diverse populations, and highlighting a need for research focused on the health implications of vitamin D that is relevant for Black families.

The developing fetus depends on maternal vitamin D transported across the placenta (Liu & Hewison, 2012). Research with animals has shown that prenatal 25(OH)D associates with neuronal differentiation, maturation, neurotransmitter synthesis, and synaptic function in the fetal brain (Cui, Gooch, Petty, McGrath, & Eyles, 2017; Kesby et al., 2010; Wagner & Hollis, 2018); neurobiological mechanisms that support behavioral, emotional and social functioning in humans. Indeed, several population-based and registry studies in Europe have reported associations between low concentrations of prenatal 25(OH)D (measured continuously and categorically) and symptoms of attention-deficit hyperactivity disorder (ADHD) among school-age children and adolescents (Daraki et al., 2018; Morales et al., 2015; Sucksdorff et al., 2021). Convergent results were reported among 6- to 13-year-olds in a large, nationwide study in the U.S. (Melough et al., 2023). Findings have been more mixed in the early childhood period (Francis et al., 2021; Keim, Bodnar, & Klebanoff, 2014; López-Vicente et al., 2019; Melough et al., 2023; Sammallahti et al., 2023), highlighting the need for a more specific focus on dimensional measures of behavioral and emotional problems (e.g. overactivity, temper tantrums, fearful behaviors) within discrete developmental windows.

Few studies have examined associations between prenatal 25(OH)D and offspring behavioral and emotional health in Black-identifying mothers and children. In one notable exception, gestational 25(OH)D was inversely related to externalizing behaviors, but unrelated to internalizing problems, in a large sample of young children aged 1.5–5 years (Melough et al., 2023). In another that included 66 toddlers identified as Black by their parent, Chawla and colleagues reported counterintuitive findings: the lowest prenatal 25(OH)D quartile was associated with fewer child internalizing problems and greater social competence (Chawla et al., 2019). Although the small sample and unequal representation of participants across quartiles were important limitations urging the need for further research, this study is noteworthy for its additional focus on prosocial child behaviors that are consistent with the theorized beneficial effects of prenatal vitamin D. Existing studies have also been limited by a lack of consideration of social-contextual factors such as stress exposure and depressive symptoms during pregnancy, which have demonstrated associations with both prenatal vitamin D status and future child health outcomes (Aghajafari, Letourneau, Mahinpey, Cosic, & Giesbrecht, 2018; Gutteling et al., 2005; Jahan et al., 2021; Morgan, Channon, Penny, & Waters, 2021; Parker, Brotchie, & Graham, 2017). Adjusting for these potential confounds is critical for clarifying the influence of prenatal vitamin D on child behavioral and emotional development.

Finally, a life-course conceptual framework, recognizing that current health is shaped by earlier exposures to environmental and psychosocial factors (Barker, 2004; Ben-Shlomo & Kuh, 2002), aligns with calls for optimizing the health and development of children well before conception (Black et al., 2022; Vaivada et al., 2022; World Health Organization, 2013). There is robust evidence that nutritional status prior to pregnancy is an important contributor to offspring health (Keenan, Hipwell, Class, & Mbayiwa, 2018; Stephenson et al., 2018). A good example is the use of folic acid supplementation to reduce the risk of neural tube defects in the fetus; the recommended timing of supplementation is prior to conception in order to achieve sufficient levels (Abate et al., 2024). Results from other nutrition-based interventions have shown that preconception, rather than prenatal, supplementation is associated with healthy offspring neurodevelopment (D'Souza et al., 2021; Hynes, Seal, Otahal, Oddy, & Burgess, 2019; Keats et al., 2021; Robinson et al., 2018). In addition, an Australian cohort study showed that higher diet quality prior to pregnancy was associated with a reduced risk of behavior problems (e.g. hyperactivity) in school-aged offspring (Gete, Waller, & Mishra, 2021), although shared-method variance from maternal reports may have contributed to these results. Despite a growing number of human studies highlighting the importance of preconception nutrition factors, we are not aware of any longitudinal investigation of the extent to which preconception levels of vitamin D associate with positive and negative child behavioral health, or indeed might enhance beneficial prenatal levels, that could help inform early (i.e. pre-pregnancy) risk assessment and interventions to support children's health.

The current prospective study sought to examine relationships between preconception and prenatal vitamin D and offspring behavioral and emotional functioning among Black-identifying families that could inform the timing of interventions to support child health. Specifically, we hypothesized that prenatal 25(OH)D concentration would be associated with fewer offspring behavioral and emotional problems and more prosocial behaviors (e.g. kindness, helpfulness) after accounting for contextual factors such as prenatal stress and depression. Given emerging evidence for the importance of preconception health, we also examined the independent effects of preconception 25(OH)D on child outcomes. Finally, we explored the extent to which preconception 25(OH)D might strengthen the association between prenatal 25(OH)D and child behavioral and emotional outcomes.

## Methods

### Sample

The sample included participants from the population-based Pittsburgh Girls Study (PGS), an ongoing longitudinal study of 2450 women originally recruited during childhood (ages 5–8 years) in 1999–2000. PGS recruitment used a random sampling process that targeted 103 238 households in Pittsburgh, PA, with oversampling of families in low-income neighborhoods (Hipwell et al., 2002; Keenan et al., 2010). More than half of PGS participants (57%, *N* = 1402) identified as Black or Black and another race. Annual PGS interviews have been conducted for over 20 years; in assessment waves 17 and 18 when participants were 21–25 years, a venous blood draw was added to the study protocol. Across the duration of the study, participant retention has been high: 87.4% of the original sample was retained at the close of annual assessment wave 20.

In 2018, PGS participants who became pregnant were invited to participate in a study examining the effects of preconception and prenatal stress on offspring neurodevelopment. In this study, enrolled participants completed a venous blood draw and self-report measures during pregnancy and follow-up assessments, including a research lab visit when the child was age 2–4 years. Approval for all study procedures was obtained from the University of Pittsburgh Human Research Protection Office. Participants provided written informed consent prior to data collection.

**Figure 1.**
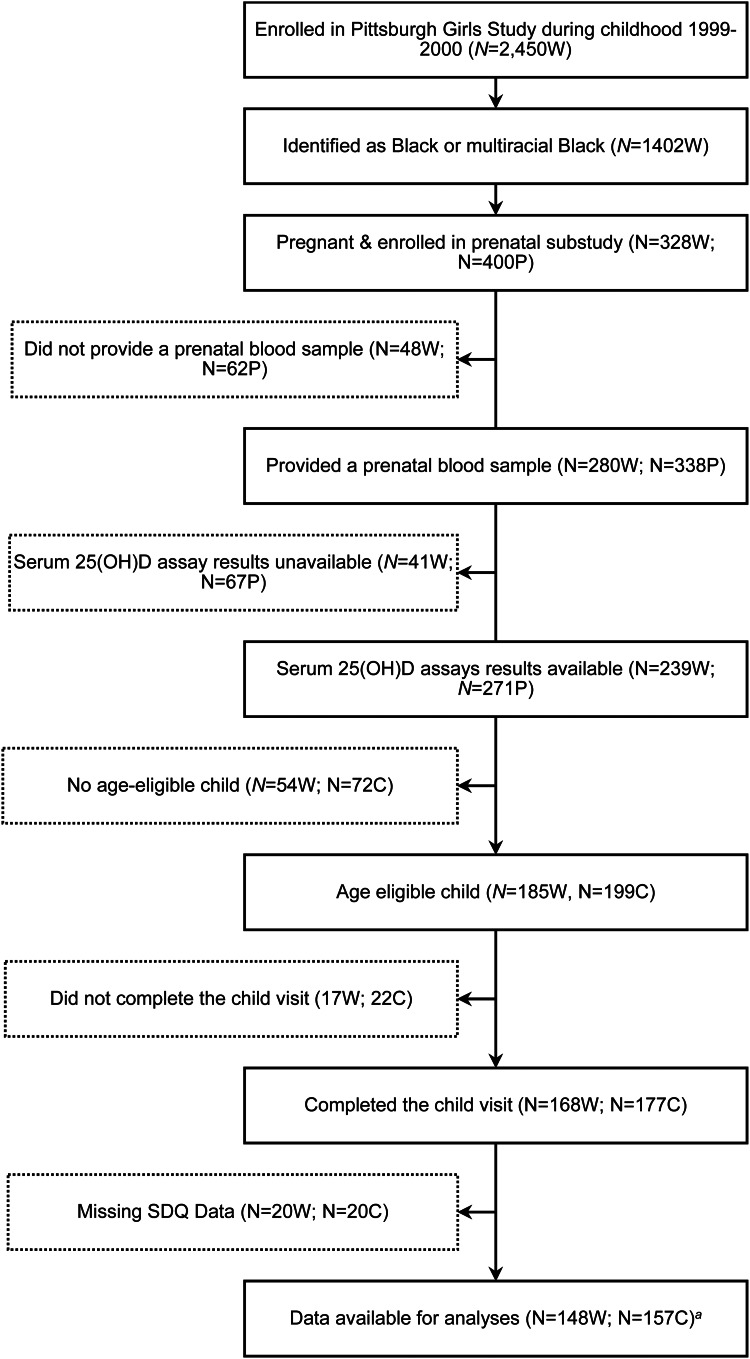
Consort diagram of the final sample. W, PGS participant; *p*, pregnancy; C, child. *^a^* One outlier was excluded from analysis resulting in a final sample of *N* = 156.

Among Black-identifying PGS participants, 328 women became pregnant at least once after 2018 and enrolled in the substudy. Of this group, 280 (85.4%) also provided a prenatal venous blood sample. At the time of analysis, prenatal serum 25(OH)D assay results were available for 239 participants. Among this group, 185 participants had one or more age-eligible children (2–4 years) enrolled in the study (*N* = 199) of whom 157 (78.9%) also had data on child health outcomes (see Fig. 1).

### Measures

#### Vitamin D concentrations

Non-fasting venous blood samples were collected from participants during the preconception and prenatal periods into silicone-coated collection tubes (Monoject 8881301512) and allowed to clot. Tubes were centrifuged at 4 °C at 1500 rpm for 30 min to induce serum separation, aliquoted into plastic vials (Fisherbrand 02-681-338), and immediately stored at −80 °C. Frozen sera were transferred on dry ice to, and subsequently assayed by the Central Ligand Assay Satellite Services Laboratory, a CAP-accredited and College of American Pathologists and Clinical Laboratory Improvement Act (CLIA)-certified lab at the University of Michigan School of Public Health. Serum samples were assayed for total 25(OH)D using an enzyme-linked immunosorbent assay (ELISA) kit according to manufacturer instructions (R&D Systems RDKAP1971). The analytical range of the 25(OH)D assay was 2.81–333 ng/ml and the average intra-assay coefficient of variation (CV) was 7.8% at 5.5 ng/ml, 5.7% at 27.4 ng/ml, and 2.5% at 81.2 ng/ml. Concentration was measured continuously and dichotomized following common dietary guidelines for sufficiency (0 = 20–49.9 ng/ml) *v.* insufficiency (1 = <20 ng/ml) (U.S. Department of Agriculture and U.S. Department of Health and Human Services, 2020).

*Child behavioral and emotional health* was measured using the parent-rated preschool version of the Strengths and Difficulties Questionnaire (SDQ; Goodman, 1997) for children aged 2–4 years. Biological mothers completed SDQ subscales assessing conduct problems, hyperactivity/inattention, emotional symptoms, peer problems, and prosocial behaviors. The five items of each subscale were rated on 3-point Likert scales as not true (0), somewhat true (1) and certainly true (2). In population samples, a three-subscale division of the preschool SDQ is recommended (Goodman, Lamping, & Ploubidis, 2010) consisting of behavioral/externalizing problems (sum of conduct problems and hyperactivity/inattention), emotional/internalizing problems (sum of emotional symptoms and peer problems) and prosocial behaviors (sum of five items, e.g. kindness, helpfulness, sharing). The SDQ preschool version has acceptable psychometric properties, including convergent validity with other measures of child behavioral and emotional problems (Croft, Stride, Maughan, & Rowe, 2015; Goodman, 1997). In the current sample, internal consistency (Cronbach's alpha) was 0.71 for behavioral problems, 0.81 for emotional problems and 0.82 for prosocial behavior. These three subscales provide indicators of behavioral and emotional health and the absence/presence of behavioral and emotional problems.

### Potential covariates

Participants reported on parity and receipt of public assistance (e.g. Special supplemental nutrition program for Women, Infants and Children [WIC], Supplemental Nutrition Assistance Program [SNAP], Temporary Assistance for Needy Families [TANF]). Given the impact of season on vitamin D synthesis, the timing of blood draws was categorized as: winter (December, January, February), spring (March, April, May), summer (June, July, August) and fall (September, October, November). Child sex assigned at birth (1 = male, 2 = female), child date of birth, birthweight, and gestational age at birth were collected from electronic birth record or by maternal report. Maternal and child ages were calculated from research visit dates relative to their respective birth dates, and gestational week of the prenatal blood draw was calculated from date of research visit and child gestational age at birth. Research visit dates were also used to determine the length of interval between the preconception and prenatal assessments.

Factors associated with both maternal 25(OH)D concentration and offspring behavioral and emotional health (Casseb, Kaster, & Rodrigues, 2019; Choy & Raine, 2022; Naugler, Zhang, Henne, Woods, & Hemmelgarn, 2013) were considered for inclusion as covariates. Preconception maternal BMI, derived from interviewer-measured height and weight, was used as a proxy for the effects of adiposity on vitamin D metabolism (Wortsman, Matsuoka, Chen, Lu, & Holick, 2000), and tobacco use (0 = no, 1 = yes) was self-reported using the Nicotine, Alcohol and Drug Use measure (Pandina, Labouvie, & White, 1984). Prenatal depression was measured by self-report using the Adult Symptom Inventory (ASRI-4; Gadow, Sprafkin, & Weiss, 2004), which assesses the frequency of DSM-IV symptoms of major depressive disorder plus two related symptoms: low self-esteem and hopelessness. Seven symptoms were rated on 4-point scales (0 = never to 3 = very often), and four symptoms (changes in appetite, sleep, activity, concentration) were scored as absent (0.5) or present (2.5) following standard scoring procedures (Gadow et al., 2004). Items were summed to form a total depression severity score. The ASRI-4 depression scale demonstrates convergent and discriminant validity and has been shown to differentiate between clinical and non-clinical samples (Gadow et al., 2004). Participants also reported on the presence/absence of life stressors (e.g. inadequate housing, financial difficulties, household conflict, exposure to violence in the community) during pregnancy using the 28-item Difficult Life Circumstances Scale (DLC; Barnard, 1994). Items were summed to indicate number of life stressors (score range = 0–28). The DLC has good psychometric properties and in pregnant samples has demonstrated predictive validity with adverse child outcomes (Curry, Campbell, & Christian, 1994).

### Analytic plan

Statistical analyses were performed with IBM SPSS 28.0 software. Potential covariates were evaluated with a correlation matrix. Study hypotheses were tested in three stepwise linear regression models with SDQ subscale scores (behavior problems, emotional problems, prosocial behavior) as the dependent variables (DVs). Prenatal 25(OH)D was entered as the primary independent variable (IV) in Step 1 after adjusting for covariates. Preconception 25(OH)D was added in Step 2 to examine possible timing effects, and the two-way interaction between preconception and prenatal 25(OH)D was added in Step 3 to examine synergistic effects on child behavioral and emotional health. We aimed to probe significant interactions using regions of significance to explore correspondence with sufficiency guidelines. A variance inflation factor ⩾1.5 was used to indicate collinearity among covariates (Kim, 2019).

## Results

### Sample characteristics

Descriptive statistics are summarized in Table 1. Participants self-identified as Black (94.2%) or Black and another race (5.8%) and had a mean age of 23.4 years (s.d. = 1.2) and 25.2 years (s.d. = 1.4) at the preconception and prenatal visits respectively. Approximately one third of participants were primiparous, 40.4% received public assistance during pregnancy, and 48.1% were in the second trimester at the time of the prenatal blood draw. The mean interval between the preconception and prenatal blood draws was 1.8 years (s.d. = 0.7) years. Participants reported on average, three life stressors (range = 0–11) during pregnancy.

**Table 1.** Characteristics of study participants (*n* = 156)

	*N* (%)	Mean (s.d.)	Range
Maternal racial identity			
Black	147 (94.2)		
Black and another race	9 (5.8)		
Preconception period:			
25(OH)D, ng/ml^a^		15.7 (7.7)	3.7–47.2
Insufficient – very low (< 12 ng/ml)	57 (38.5)		
Insufficient – low (12–19.9 ng/ml)	50 (33.8)		
Sufficient (20–49.9 ng/ml)	41 (27.7)		
High (> 50 ng/ml)	0		
Season of preconception blood draw			
Winter (December, January, February)	35 (23.6)		
Spring (March, April, May)	12 (8.1)		
Summer (June, July, August)	72 (48.6)		
Fall (September, October, November)	29 (19.6)		
Maternal age at blood draw, years		23.4 (1.2)	21.0–25.9
Receipt of public assistance	43 (27.7)		
Body Mass Index (BMI)		29.3 (8.3)	16.9–60.2
Past year smoking status	44 (28.4)		
Highest level of education			
No high school diploma	13 (8.8)		
General Educational diploma (GED)	11 (7.5)		
High school diploma	83 (56.5)		
Associate degree or training certificate	34 (23.1)		
Bachelor's degree	6 (4.1)		
Prenatal period:			
25(OH)D, ng/ml		18.0 (9.2)	5.7–62.7
Insufficient – very low (< 12 ng/ml)	41 (26.3)		
Insufficient – low (12-19.9 ng/ml)	66 (42.3)		
Sufficient (20–49.9 ng/ml)	47 (30.1)		
High (> 50 ng/ml)	2 (1.3)		
Season of prenatal blood draw			
Winter (December, January, February)	37 (23.7)		
Spring (March, April, May)	34 (21.8)		
Summer (June, July, August)	42 (26.9)		
Fall (September, October, November)	43 (27.6)		
Maternal age at blood draw, years		25.2 (1.4)	22.4–28.3
Primiparous	55 (35.7)		
Prenatal gestation, weeks		20.3 (8.9)	6.0–38.9
Prenatal trimester			
First (1–12 weeks)	43 (27.6)		
Second (13–26 weeks)	75 (48.1)		
Third (27+ weeks)	38 (24.4)		
Prenatal depression severity		7.8 (4.3)	0–27
Life stressors		3.3 (2.6)	0 −11
Public assistance	63 (40.4)		
Preconception to prenatal periods:			
Change (*Δ*) in 25(OH)D, ng/ml		2.1 (8.9)	−19.2–42.7
Interval between blood draws. years		1.8 (0.7)	0.4–3.8
Early childhood:			
Child age at assessment, years		2.8 (0.5)	2.0–4.0
Child sex, female	83 (53.2)		
Child birthweight, g^b^		3016 (570)	680–4390
Gestational age at birth, weeks		38.6 (2.4)	26.3–41.6
Behavior problems		6.7 (3.2)	0–15
High/very high	34 (25.6)		
Emotional problems		3.1 (3.3)	0–16
High/very high	43 (28.5)		
Prosocial behaviors		6.3 (2.7)	0–10
Low/very low	59 (37.8)		

*Note:* a Preconception 25(OH)D data were missing for eight participants; b Birthweight was missing for 15 children.

Mean serum 25(OH)D concentrations were 15.5 ng/ml (s.d. = 7.7) before pregnancy and 18.0 ng/ml (s.d. = 9.2) during pregnancy; levels designated as insufficient according to U.S. Department of Agriculture and U.S. Department of Health and Human Services (2020) guidelines. In the preconception period, 72.3% of participants had insufficient 25(OH)D concentrations (<20 ng/ml), and during pregnancy the proportion was 68.6%. An outlier with vitamin D concentrations exceeding eight standard deviations above the mean was excluded to avoid disproportionate influence on analyses (Chambers, Hentges, & Zhao, 2004). The overall mean increase from preconception to pregnancy was very small (2.1 ng/ml, s.d. = 8.9), but there was considerable variability in the extent and direction of change in 25(OH)D over time (range: −19.2 to 42.7 ng/ml).

Offspring (53% female) completed a research visit on average at 2.8 years (s.d. = 0.5). Mean gestational age at birth was 38.6 weeks (s.d. = 2.4) and 12.8% of infants were born preterm (< 37 weeks). Mean birthweight was 3016 g (s.d. = 570); 11.3% had a birthweight < 2500 g, including two infants with birthweight < 1000 g. Child birthweight and gestational age at birth were highly correlated (*r* = 0.76, *p* < 0.001), and as birthweight was missing for 15 participants, we excluded it from further analyses. Maternal reports of child behavioral and emotional problems were similar to other community samples: 25.6% of children scored in the high/very high range for behavioral problems, 28.5% scored in the high/very high range for emotional problems, and 37.8% in the low/very low range for prosocial behavior (http://sdqinfo.org).

Concentrations of 25(OH)D before and during pregnancy were moderately stable (*r* = 0.48, *p* < 0.001) even after adjusting for the length of the interval between the two blood draws (partial *r* = −0.31, *p* < 0.001). As shown in Table 2, preconception 25(OH)D (continuously measured) was significantly and negatively correlated with prenatal life stressors and child behavior problems. There were no associations between sufficient/insufficient levels in the preconception period and other study variables. Prenatal 25(OH)D was higher in first-time mothers, was negatively associated with depression severity and with offspring behavioral and emotional problems and was positively associated with child prosocial behavior. Prenatal insufficiency showed a similar pattern of associations with child outcomes. Primiparous mothers reported higher levels of behavior problems in their child and fewer prosocial behaviors. Older child age was associated with more positive child behavior (i.e. fewer emotional problems and more prosocial behavior). Additionally, prenatal depression and life stress were positively correlated with child behavioral problems; prenatal depression was also associated with child emotional problems. Finally, offspring behavioral and emotional outcomes showed small to moderate correlations with each other, indicating that they were related but distinct domains of functioning

**Table 2.** Bivariate correlations among study variables

		1	2	3	4	5	6	7	8	9	10	11	12	13	14	15	16	17
1	Preconception 25(OH)D	–																
2	Preconception insufficiency	−0.79**	–															
3	Public assistance	0.11	−0.06	–														
4	BMI	0.10	−0.10	−0.05	–													
5	Smoking	0.10	−0.05	−0.04	−0.13	–												
6	Prenatal 25(OH)D	0.48**	−0.39**	−0.02	0.05	−0.07	–											
7	Prenatal insufficiency	−0.53**	0.49**	−0.01	−0.10	0.09	−0.74**	–										
8	Season in pregnancy	−0.06	0.03	−0.11	−0.03	−0.01	0.11	−0.05	–									
9	Maternal age	0.01	−0.01	0	0.04	0.04	−0.03	−0.01	0.09	–								
10	Primiparity	0.07	−0.08	−0.46**	0.12	0.06	0.16*	−0.10	0.01	−0.13	–							
11	Prenatal depression	−0.01	−0.07	0.01	−0.08	0.17*	−0.16*	0.10	0.01	−0.02	0.06	–						
12	Life stressors	−0.22**	0.15	−0.08	0.08	0.04	−0.06	0.02	0.06	0.18*	0.04	0.31**	–					
13	Child age	0.04	−0.01	−0.01	−0.01	−0.19*	0.10	−0.10	0.12	−0.17*	−0.08	0.03	0.01	–				
14	Female child sex	0.14	−0.13	−0.06	−0.01	0.07	0.16*	−0.16*	−0.11	0.03	−0.01	−0.10	0.02	−0.09	–			
15	Gestational age at birth	0.01	0.04	0.01	0.01	−0.01	0.03	0.03	0.16*	−0.06	−0.02	0.08	−0.06	0.04	0.09	–		
16	Behavior problems	−0.17*	0.07	−0.05	0.16	0.06	−0.21**	0.17*	0.02	−0.06	0.19*	0.30**	0.16*	−0.03	−0.11	0.05	–	
17	Emotional problems	−0.08	−0.05	−0.02	0.16	0.12	−0.18*	0.16*	0.10	0.09	0.09	0.19*	0.05	−0.16*	−0.09	−0.03	0.57**	–
18	Prosocial behaviors	0.11	−0.08	0.11	0.01	−0.06	0.27**	−0.25**	0.06	−0.01	−0.16*	−0.10	−0.02	0.20*	0.09	0.05	−0.36**	−0.27**

*Note*: ** *p* < 0.01; * *p* < 0.05.

Given their associations with maternal 25(OH)D at either timepoint and/or the DVs, primiparity, prenatal depression, life stressors, child sex assigned at birth, and child age were selected for inclusion as covariates in the regression models. Other potential covariates identified in prior research (preconception BMI and smoking status, season, prenatal age, child gestational age at birth and receipt of public assistance) were excluded given their lack of association with either the IVs or DVs in the current study.

### Associations between prenatal and preconception 25(OH)D and behavioral and emotional outcomes in early childhood

Results of stepwise linear regression analyses examining prospective associations between prenatal and preconception 25(OH)D and later child behavioral and emotional problems and prosocial behavior are shown in Table 3. Model statistics indicated reliable prediction of behavior problems in each step with modest effect sizes (*R*^2^ = 0.16–0.17). Prenatal 25(OH)D was inversely related to child behavior problems (*β* = −0.19, 95% CI −0.12 to −0.01) after adjusting for primiparity, prenatal depression severity, life stressors, child age, and child sex. Preconception 25(OH)D explained no additional variance in child behavior problems when added in Step 2, and attenuated the positive association between prenatal 25(OH)D and behavior problems to non-significance. These results were unchanged when the two-way interaction between preconception and prenatal 25(OH)D was added to the model in the final step.

**Table 3.** Prospective associations between prenatal and preconception 25(OH)D and child behavioral and emotional outcomes

	Behavior problems				Emotional problems				Prosocial behavior			
	Step 1		Step 2		Step 1		Step 2		Step 1		Step 2	
	*β*	95% CI	*β*	95% CI	*β*	95% CI	*β*	95% CI	*β*	95% CI	*β*	95% CI
(Intercept)	–	3.13–9.72	–	3.37–10.04	–	2.67–9.65	–	2.61–9.72	–	−0.06 to 5.50	–	−0.02 to 5.63
Prenatal 25(OH)D	**−0.19**	**−0.12 to −0.01**	−0.14	−0.11 to 0.01	−0.14	−0.11 to 0.01	−0.14	−0.12 to 0.02	**0.27**	**0.03–0.12**	**0.28**	**0.03–0.13**
Preconception 25(OH)D	–	–	−0.09	−0.11 to 0.04	–	–	−0.01	−0.08 to 0.08	–	–	−0.03	−0.08 to 0.05
Primiparous	**0.19**	**0.27–2.30**	**0.19**	**0.27–2.30**	0.09	−0.44 to 1.72	0.09	−0.44 to 1.72	**−0.19**	**−1.89 to −0.17**	−0.19	−1.89 to −0.17
Prenatal depression	**0.23**	**0.06–0.29**	**0.25**	**0.06–0.30**	0.16	−0.01 to 0.24	0.16	−0.01 to 0.25	−0.04	−0.13 to 0.07	−0.04	−0.13 to 0.08
Life stressors	0.07	−0.11 to 0.28	0.05	−0.15 to 0.26	−0.01	−0.22 to 0.20	−0.01	−0.23 to 0.21	0.01	−0.16 to 0.18	0.01	−0.17 to 0.18
Child age	−0.01	−0.97 to 0.88	−0.01	−0.97 to 0.88	−0.15	1.91–0.04	−0.15	−1.92 to 0.05	**0.16**	**0.04–1.60**	**0.16**	**0.04–1.60**
Child sex	−0.06	−1.33 to 0.61	−0.05	−1.29 to 0.66	−0.06	−1.45 to 0.61	−0.06	−1.45 to 0.62	0.06	−0.53 to 1.11	0.06	−0.52 to 1.13

*Note*: *β*, standardized coefficient; 95% CI, 95% Confidence interval. Significant findings bolded for emphasis. Model results were unchanged with the addition of the two-way preconception × prenatal 25(OH)D interaction in Step 3.

Model summary: *Behavior problems*: Step 1: *R*^2^ = 0.16, *F*_(6,149)_ = 4.74, *p* < 0.001; Step 2: *R*^2^ = 0.17, *F*_(7,148)_ = 4.22, *p* < 0.001; Step 3: *R*^2^ = 0.17, *F*_(8,147)_ = 3.67, *p* < 0.001*; Emotional problems*: Step 1: *R*^2^ = 0.09, *F*_(6,149)_ = 2.53, *p* < 0.05; Step 2: *R*^2^ = 0.09, *F*_(7,148)_ = 2.15, *p* < 0.05; Step 3: *R*^2^ = 0.10, *F*_(8,147)_ = 1.93, *ns; Prosocial behaviors*: Step 1: *R*^2^ = 0.14, *F*_(6,149)_ = 4.08, *p* < 0.01; Step 2: *R*^2^ = 0.14, *F*_(7,148)_ = 3.50, *p* < 0.01; Step 3: *R*^2^ = 0.16, *F*_(8,147)_ = 3.44, *p* = 0.001. Variance Inflation Factors ranged from 1.03 to 1.41.

Step 1 results remained the same in sensitivity analyses that excluded eight participants without preconception 25(OH)D data. Results were also unchanged if the two extremely low birth weight infants were excluded.

Results showed no association between prenatal 25(OH)D in Step 1 and child emotional problems after adjusting for covariates. Similarly, preconception 25(OH)D explained no additional variance in emotional problems when added to the model in Step 2. In addition, results showed no synergistic effects of preconception × prenatal 25(OH)D on later child emotional problems.

When prosocial behavior was examined as the outcome, results revealed a significant positive association between prenatal 25(OH)D concentration and positive child behavior (*β* = 0.27, 95% CI 0.03–0.12) after accounting for covariates in the adjusted model. This positive association remained in Step 2 (*β* = 0.28, 95% CI 0.03–0.13), after also accounting for 25(OH)D concentration in the preconception period. There was no unique association between preconception 25(OH)D and early childhood prosocial behavior after accounting for prenatal 25(OH)D and covariates, and no interaction between preconception and prenatal 25(OH)D on child prosocial behavior.

## Discussion

Leveraging data from a multi-method longitudinal study, we investigated cross-generational health implications of maternal vitamin D in a sample of Black mothers and their children living in the northeast region of the U.S. At the bivariate level, findings showed consistent evidence of associations between prenatal serum 25-hydroxyvitamin D concentrations and positive child behavior (i.e. lower behavioral and emotional problems, higher levels of prosocial behavior) in early childhood, and some indication of an association with child behavior problems from maternal 25(OH)D levels measured in the years prior to pregnancy. However, after adjusting for covariates, results highlighted the importance of 25(OH)D in the prenatal period and, in the case of prosocial behavior, even after accounting for stability in 25(OH)D levels across the transition to pregnancy.

These results build on the existing literature in several critical ways. First, even though mean levels of prenatal and preconception 25(OH)D among study participants were generally low according to commonly used dietary guidelines (U.S. Department of Agriculture and U.S. Department of Health and Human Services, 2020), there was a consistent positive pattern of association between prenatal levels and offspring behaviors in terms of fewer behavior problems (conduct problems and hyperactivity/inattention) as well as higher levels of prosocial behavior (e.g. kindness toward younger children). This inverse association with behavior problems is similar to results reported for samples (predominantly White) with higher mean 25(OH)D levels during pregnancy (Daraki et al., 2018; Francis et al., 2021; Morales et al., 2015; Sucksdorff et al., 2021) suggesting that beneficial effects may exist across the concentration continuum. Furthermore, these results were robust after adjusting for several maternal and child sociodemographic variables including the potentially confounding effects of prenatal depressive symptoms and life stress. Indeed, higher prenatal 25(OH)D concentration was associated with lower depression severity, as has been shown previously (Aghajafari et al., 2018), suggestive of neuroregulatory effects of vitamin D (de Abreu, Eyles, & Feron, 2009; McCann & Ames, 2008) or health behavior differences (Kris-Etherton et al., 2021). Given that prenatal depression and stress showed expected associations with later child behavioral problems (Gutteling et al., 2005; Morgan et al., 2021), adjusting for prenatal depression and life stress was important for improving the precision of our model.

Second, despite moderate correlation between behavioral and emotional problems, our results suggested an outcome-specific effect: prenatal 25(OH)D was related to offspring behavioral but not emotional problems in adjusted models. Similar results were reported in a large US-based study (Melough et al., 2023) in which prenatal 25(OH)D was associated only with externalizing, and not with internalizing behaviors. Understanding this specific outcome effect in toddlerhood will be especially important for identifying early developmental pathways to psychopathology in childhood and adolescence (Brennan, Shaw, Dishion, & Wilson, 2012; Roza, Hofstra, Van Der Ende, & Verhulst, 2003; Wiggins, Mitchell, Hyde, & Monk, 2015) that could inform preventive interventions. The current study finding is not readily explained by differences in the variability of SDQ subscale scores, or the overall prevalence of emotional, relative to behavioral, disorders among preschool children (Egger & Angold, 2006). It is possible that this differential association may reflect specific pathophysiology or a clustering of structurally embedded adversity factors that are more consistently associated with child behavioral problems, and which may also contribute to differences in prenatal vitamin D dietary intake and supplementation (Bowen, Elliott, & Hardison-Moody, 2021). Further research is warranted to determine whether this pattern of results is robust, particularly given some evidence for associations between prenatal 25(OH)D and infant negative affectivity (Sammallahti et al., 2023), a transdiagnostic risk factor for both behavioral and emotional problems later in childhood.

Third, results from this longitudinal study offer some limited support for the notion that the pre-pregnancy period is a potential window of opportunity for child health promotion (Keenan et al., 2018). Our findings showed that higher preconception 25(OH)D concentration was associated with fewer child behavior problems when examined at the bivariate level. However, after also accounting for prenatal concentrations in adjusted models, the results showed no unique association between preconception 25(OH)D and child behavior problems. Furthermore, the inclusion of *both* preconception and prenatal 25(OH)D in this model attenuated the previously observed association between prenatal levels and behavior problems, which may have related to patterns of stability in 25(OH)D spanning the period from preconception to pregnancy. The extent of 25(OH)D stability, however, does not negate the potential importance of nutrition status *before* pregnancy for child behavioral health; effects that may be mediated by healthy conditions for oocyte maturation and implantation and epigenetic imprinting (Gete et al., 2021) although much work is still needed to elucidate proximal neurobiological mechanisms. Given that there was considerable heterogeneity in the extent and direction of change in 25(OH)D levels between preconception and pregnancy, modeling trajectories of change could be a useful next step to explore these results. Finally, our findings provided no indication that preconception 25(OH)D concentration might enhance the associations between prenatal levels and offspring behavioral and emotional outcomes, although the current sample may have been underpowered to detect these effects and larger samples are needed in future work.

### Limitations

The current study benefitted from a unique longitudinal design that began before pregnancy, high participant retention and rigorous measures, but several limitations should be considered along with these strengths. The results may not generalize to individuals identifying as other than Black race or families living in other regions of the U.S. Because we had limited information on other dietary factors (e.g. beneficial fatty acids) and nutrition supplementation, we cannot rule out the possibility that other aspects of diet account for the effects observed here. In addition, lower concentrations of maternal 25(OH)D could be indicative of food insecurity, limited socioeconomic resources, and access to quality health care. While adjusted models included several covariates (e.g. life stressors, depression) that are related to these factors, there may have been residual confounding by unmeasured influences. Furthermore, although the direction of association between consumption of alcohol and vitamin D is unclear (Tardelli, do Lago, da Silveira, & Fidalgo, 2017), prenatal alcohol use may have been an additional source of confounding in the current study. Participants were assessed at different stages of pregnancy depending on when information became available, and so the timing of prenatal 25(OH)D measurement varied. Given that the first and second trimesters are critical for developing fetal cortical structures related to behavioral regulation (Rice & Barone, 2000), effect estimates may have been reduced by including participants assessed later in pregnancy even though circulating levels of vitamin D tend to be relatively stable across pregnancy (Hauta-Alus et al., 2018). While the current sample size was too small to test this hypothesis with systematic exclusion of participants by trimester, future work could ascertain timing and variability in exposures vis-à-vis fetal development. In the current study, offspring outcomes were also assessed at a single timepoint, and early in development. Continued follow-up is needed to elucidate the extent to which preconception and/or prenatal 25(OH)D could have a lasting positive influence on behavioral development. Finally, offspring outcomes were assessed by maternal report, which may differ from that of preschool carers or health-care professionals.

## Conclusion

Prospective research examining associations between preconception and prenatal nutritional factors and behavioral and emotional outcomes in offspring is critical for informing the type and timing of early interventions designed to support healthy development. Given that Black families are often underrepresented in clinical research, and that structural inequities (e.g. systemic racism, residential segregation, access to affordable food, quality health care) are known contributors to both prenatal nutrition status and child health (Parker, Tovar, McCurdy, & Vadiveloo, 2020), there is an urgent need for studies with adequate representation of Black Americans to identify relevant, modifiable targets that support positive health and could ultimately reduce health disparities. The importance of this work is further underscored by recent evidence that vitamin D may buffer the negative effects of early adversity on child health (Choy & Raine, 2022). Although awareness of the importance of preconception nutrition for maternal and child health has increased in recent years, there is substantial untapped potential for a positive public health impact even in the absence of pregnancy planning (Stephenson et al., 2018). Our results, showing relative preconception-to-pregnancy stability and associations between maternal 25(OH)D and more positive behavior in early childhood, support this notion and highlight an opportunity for population-level, universal interventions that could be implemented in the reproductive years.

**Key points**

- Low prenatal vitamin D (25[OH]D) is linked to behavioral problems in school-aged children. However, research is lacking in Black families and contextual factors like stress and depression are often not accounted for.
- Theory and emerging data suggest that nutritional status prior to pregnancy may be important for offspring behavioral and emotional health.
- In this prospective study of Black mothers and children, higher concentrations of 25(OH)D associated with fewer preschooler behavior problems and more prosocial behavior in adjusted models. Results revealed no synergistic effects of timing.
- Results highlight potential opportunities for early nutrition interventions (before and during pregnancy) to support healthy child development.
